# The liposomal delivery of hydrophobic oxidovanadium complexes imparts highly effective cytotoxicity and differentiating capacity in neuroblastoma tumour cells

**DOI:** 10.1038/s41598-020-73539-6

**Published:** 2020-10-07

**Authors:** Elsa Irving, Aristides D. Tagalakis, Ruhina Maeshima, Stephen L. Hart, Simon Eaton, Ari Lehtonen, Andrew W. Stoker

**Affiliations:** 1 Great Ormond Street Institute of Child Health, University College London, 30 Guilford Street, London, WC1N 1EH UK; 2 Department of Chemistry, University of Turku, 20014 Turun yliopisto, Finland; 3 Present Address: Department of Biology, Edge Hill University, Ormskirk, L39 4QP UK

**Keywords:** Biochemistry, Cell biology, Cancer, Cancer therapy, Paediatric cancer, Drug delivery

## Abstract

Oxidovanadium complexes with organic ligands are well known to have cytotoxic or differentiating capabilities against a range of cancer cell types. Their limited use in clinical testing though has resulted largely from uncertainties about the long-term toxicities of such complexes, due in part to the speciation to vanadate ions in the circulation. We hypothesised that more highly stable complexes, delivered using liposomes, may provide improved opportunities for oxidovanadium applications against cancer. In this study we sourced specifically hydrophobic forms of oxidovanadium complexes with the explicit aim of demonstrating liposomal encapsulation, bioavailability in cultured neuroblastoma cells, and effective cytotoxic or differentiating activity. Our data show that four ethanol-solubilised complexes with amine bisphenol, aminoalcohol bisphenol or salan ligands are equally or more effective than a previously used complex bis(maltolato)oxovanadium(V) in neuroblastoma cell lines. Moreover, we show that one of these complexes can be stably incorporated into cationic liposomes where it retains very good bioavailability, apparently low speciation and enhanced efficacy compared to ethanol delivery. This study provides the first proof-of-concept that stable, hydrophobic oxidovanadium complexes retain excellent cellular activity when delivered effectively to cancer cells with nanotechnology. This offers the improved prospect of applying oxidovanadium-based drugs in vivo with increased stability and reduced off-target toxicity.

## Introduction

There has been interest over many decades in the use of vanadium-derived complexes as pharmacological agents to treat human disease, most notably in diabetes and cancer. There are numerous models of such complexes having anti-cancer and anti-diabetic effects, both in vitro and in animals^1–5^, supporting their potential for therapeutic development in humans. However, although metallotherapeutics research with vanadium is long-established, there have been limited clinical trials. Such trials indicated that vanadyl sulphate and the oxidovanadium complex bis(ethylmaltolato)oxovanadium(IV) (BEOV) could be well tolerated. Nevertheless, the reluctance to pursue further trials is based on the potential oxidative and genotoxic effects of high dose vanadium and the fear of cumulative, long-term toxicities in off-target tissues^2,6,7^. This has certainly generated doubts about applicability for chronic diseases such as diabetes^8,9^. However, these issues may be of lesser concern for cancer patients undergoing short-term treatments. This has encouraged continued investigation of improved vanadium derivatives with anti-cancer potential^10–12^. In addition, more recent technologies make it increasingly possible to target drug molecules more precisely to tumour tissues, ameliorating off-target problems. This may allow vanadium complexes to remain as a potential therapeutic platform.

Vanadium exists in oxidation states − III to + V, but in biological solutions the majority is in the form of vanadyl cations (IV) or vanadate ions (V)^13^. Orthovanadate has a tetra-coordinated structure with the vanadate ion at the centre, similar to a phosphate group. Because of this, vanadate is a broad specificity and reversible inhibitor of protein tyrosine phosphatase (PTP) enzymes^5,14,15^. This is considered one of its major biochemical effector mechanisms and PTPs are key players in many signalling pathways that contribute to the onset and progression of cancer^16–18^. Nevertheless, the similarity to phosphate means that vanadate can accumulate in tissues with high phosphate content, such as bone^3,4,19,20^. One approach for tackling this issue has been the development of oxidovanadium complexes with a wide range of organic ligands. These include maltolato vanadium complexes, vanadocenes, and peroxidovanadium complexes^3^. Such complexes do not mimic phosphate and are commonly too large to act as PTP inhibitors themselves, but they have improved oral bioavailability in vivo compared to vanadate, effectively permitting better vanadium absorption by the gut and release into the circulation^4,21^. In some cases, the oxidovanadium complexes are also made with the intention of delivering toxic ligands. However, all of these complexes are subject to rapid ligand exchange and speciation in the gut or circulation, releasing or rearranging the ligands and generating vanadium complexation with proteins such as albumin or transferrin, or with citrate and ascorbate, before reaching target cells^9,10,22–28^. This common instability of oxidovanadium complexes and release of circulating vanadate is considered a potential hindrance to their clinical application. Therefore, to harness better the anti-cancer properties of vanadium, it would be advantageous to consider carefully the choice of more stable complexes, and how they could be delivered to cells with minimal speciation.

We have hypothesised that highly hydrophobic oxidovanadium complexes may offer better stability in aqueous environments. This is supported by studies of Reytman and co-workers who showed that diamino tris(phenolato) liganded vanadium ions can generate hydrophobic and stable complexes with good anti-cancer activity in HT-29 colon carcinoma cells^29^. We further hypothesised that hydrophobicity would offer an alternative delivery route for such complexes using nanotechnology. Nanocomposite carriers containing metavanadate and chitosan have been used in mouse models of type 2 diabetes^30,31^, while polyethylene glycol (PEG) lipid micelles containing vanadium disulphide nanodots displayed tumour uptake in mice for image-guided photo thermal cancer therapy^30–32^. Although at the time of our study there were no examples of nanotechnology being used to deliver larger oxidovanadium complexes, we considered that liposomes would offer a good delivery vehicle due to the hydrophobicity of the complexes. Liposomes have already been proven to successfully deliver both hydrophilic and hydrophobic molecules to cancer cells^33,34^ and liposomes could also provide protection against oxidovanadium complex speciation in the bloodstream as well as targeting possibilities in solid tumours. Once delivered into cells in this manner, we would nevertheless still expect intracellular speciation to occur.

Our previous studies demonstrated that the complex bis(maltolato)oxovanadium(IV) (BMOV) is both cytotoxic and differentiative in neuroblastoma tumour-derived cell lines^35,36^. Since BMOV is water soluble and not particularly stable in aqueous environments, our aim was to identify alternative, hydrophobic oxidovanadium complexes and to demonstrate for the first time their cytotoxicity in neuroblastoma tumour cells when delivered with liposomes. Here we demonstrate this approach using a class of highly hydrophobic V(V) complexes with aminoalcohol bisphenol and salan ligands that we have described previously^37–39^. Our data confirm the cytotoxicity and differentiating capacity of each compound in several neuroblastoma cell lines and, moreover, we successfully deliver one of these complexes to cultured neuroblastoma tumour cells using liposomes, where it retains good bioavailability and efficacy.

## Results

### Cytotoxicity of hydrophobic oxidovanadium complexes compared to BMOV

Oxidovanadium complexes with organic ligands are effective anti-cancer agents in experimental models, including neuroblastoma^2,3,5^. Most of these complexes are water soluble, such as BMOV, and in aqueous environments such as culture media or serum can be subject to dissolution into their component parts of vanadate and soluble ligands^10,22–25^. Our first aim was to identify complexes that are water insoluble and retain similar properties to BMOV in being effective as cytotoxic and differentiative agents in neuroblastoma cells. Such complexes would then be evaluated for liposomal delivery.

**Figure 1:**
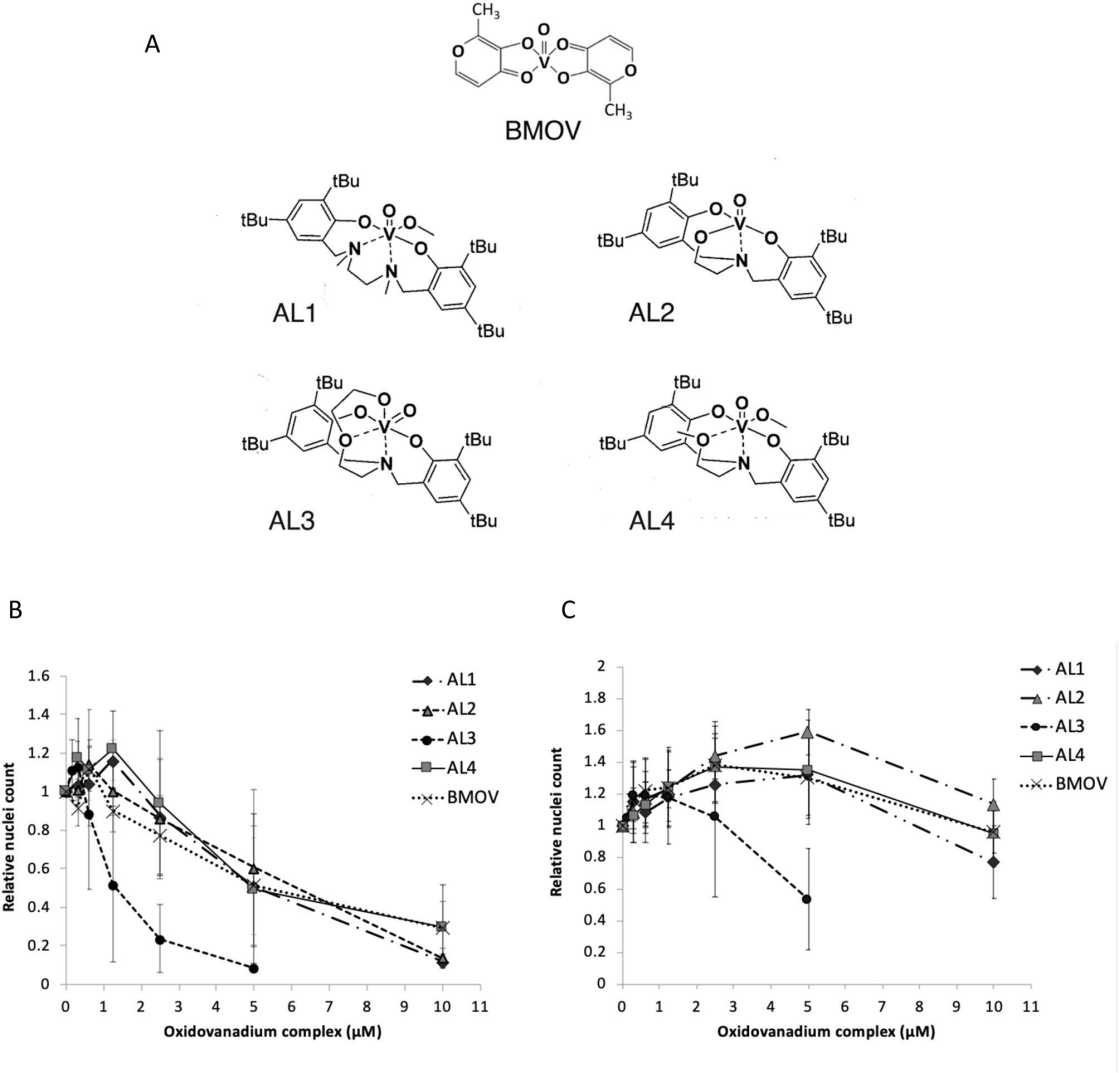
Structures and cytotoxicity of oxidovanadium complexes. (**A**) Structures of oxidovanadium complexes. AL1 has a diamine bis (phenolato) tetradentate ligand (a salan), AL2–4 have aminoalcohol bisphenol ligands, and all have coordinated V(IV) ions. AL1–4 are highly water insoluble. BMOV has maltolato ligands, a coordinated vanadium(V) ion and is water soluble. (**B**,**C**) cytotoxicity of oxidovanadium complexes in IMR32 and KELLY cells. IMR32 (**B**) and KELLY (**C**) cells were treated with increasing concentrations of oxidovanadium complexes up to 10 µM for BMOV and AL1,2,4 and up to 5 µM for AL3. After 3 days, cells were stained with Hoechst and nuclei were counted and normalised to ethanol-only treated cells (n = 3, SD shown).

We have previously prepared a series of amine bisphenol oxidovanadium complexes (AL2–AL4 in Fig. 1), which are highly insoluble in aqueous solutions^37–39^. A salan complex AL1 is also highly insoluble in water^38^. While analogous to iso-propanolate derivative L^7^VO(OiPr), which had previously been shown to have little cytotoxicity in OVCAR01 or HT29 tumour cells^40^, we wished to assess AL1 in neuroblastoma cells. The aminoalcohol bisphenolate complexes, AL2 and AL3 or amine bisphenolate complex AL4 have not been tested on cells before.

The compounds were very poorly soluble in DMSO and so we assessed the cytotoxic potential of AL1–4 against neuroblastoma-derived tumour cell lines when delivered in sub-toxic levels of ethanol. Cytotoxicity was then compared to BMOV^35,36^. Maximal solubilities in ethanol ranged from 500 µM (AL3) to 10 mM (AL1,2,4), producing distinctly coloured solutions that were very stable at room temperature. Neuroblastoma cells were treated with 5 µM or 10 µM of AL1, AL2 and AL4 for 3 days. Due to its particularly poor solubility, AL3 was used at 2.5 µM and 5 µM. Treated IMR32 and KELLY cells showed clear evidence of cytotoxicity, with initially a poorly adhesive morphology preceding the appearance of apoptotic or necrotic cell bodies, similar to that seen previously with BMOV^35^. The morphological cytotoxicity of the oxidovanadium complexes was lower in KELLY than in IMR32, with poorly-adherent KELLY cells surviving better and possibly increasing cell numbers at low chemical doses (Supplementary Fig. S1). All of the hydrophobic oxidovanadium complexes appeared equally or more effective as equivalent doses of BMOV. The 5 µM AL3-treated cells experienced qualitatively greater cytotoxicity compared with the other complexes at 5 µM, more similar to their 10 µM doses. Surviving cells were then quantified using Hoechst for nuclear counting. This Hoechst method was more accurate than MTS-based metabolic assays, since oxidovanadium complexes interfered with the MTS metabolic readout (data not shown). The data reveal again that all complexes except AL3 had similar potency as BMOV (Fig. 1B,C). We again noted the growth stimulation at low doses, but the basis for this biphasic response remains unclear. When comparing EC_50_ values, AL3 is at least 2.4-fold more potent than other complexes in KELLY cells, and at least 3.7-fold more potent in IMR32 cells (Table 1; Supplementary Fig. S2). We conclude that these four complexes can be delivered in ethanol-solubilised forms to cultured neuroblastoma cells, where they evidence similar, or in the case of AL3 significantly greater, cytotoxicity compared to BMOV.

**Table 1:** Mean EC_50_ calculations from cell survival curves generated for Fig. 1B,C (n = 3).

Complex	IMR32 EC_50_ µM	KELLY EC_50_ µM
BMOV	5.30	16.60^a^
AL1	5.10	12.40^a^
AL2	6.10	16.90^a^
AL3	1.35	5.15^a^
AL4	5.00	15.45^a^

Cells were treated for 3 days with oxidovanadium complexes before assaying cell numbers.

^a^Estimated values only for KELLY, generated through extrapolation of Fig. 1C curves to reach the 50% survival point.

### Glutathione suppression enhances cytotoxicity

**Figure 2:**
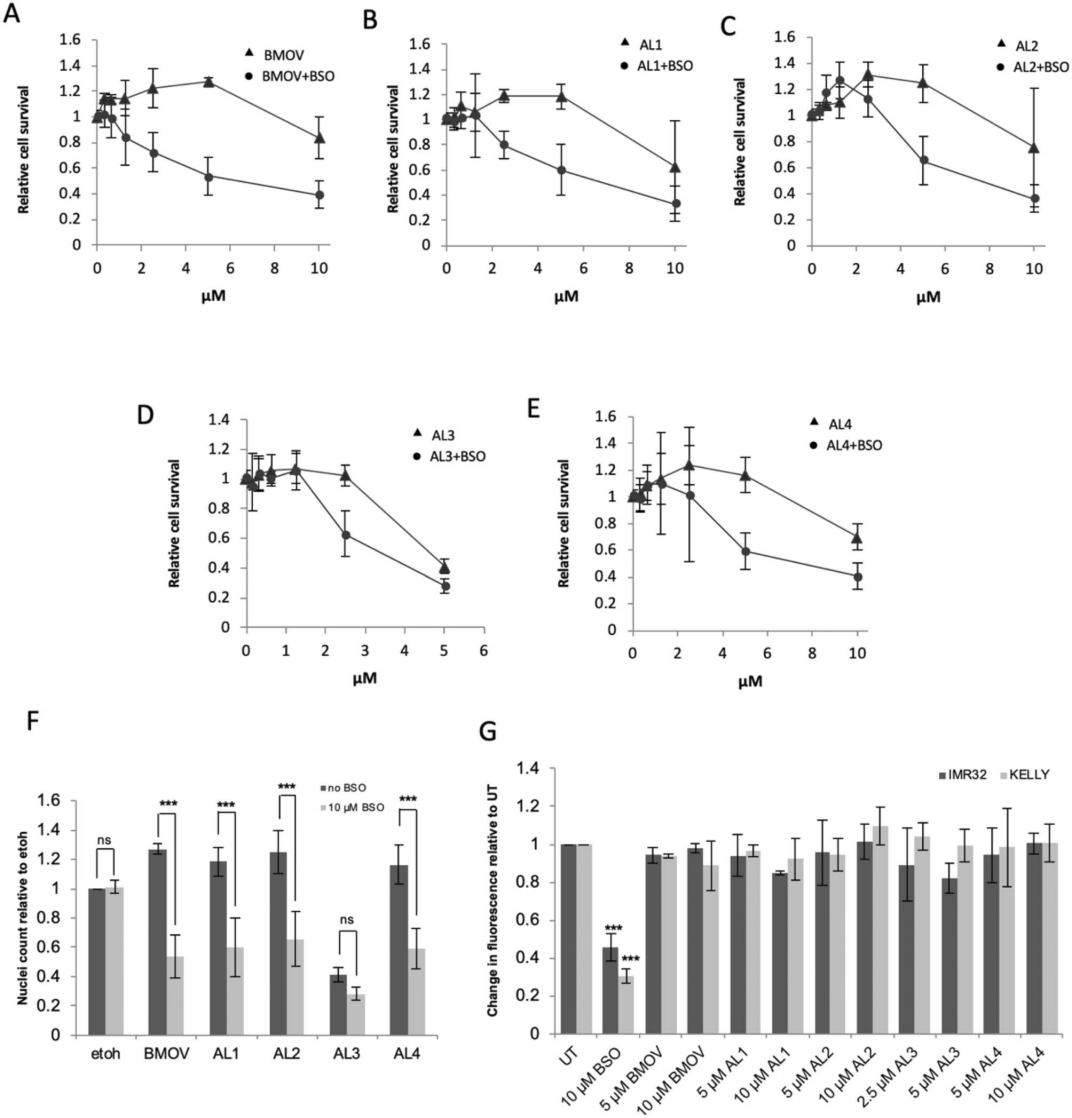
The relationship of glutathione dynamics to cytotoxicity and ROS production. (**A–E**) BSO treatment enhances oxidovanadium complex-induced cytotoxicity in neuroblastoma cells. KELLY cells were treated with increasing concentrations of oxidovanadium complexes (up to 10 µM for BMOV and AL1,2,4, and 5 µM for AL3) with and without 10 µM BSO for 3 days, after which surviving cells were quantified using nuclei staining and normalised to ethanol-only values (n = 3). (**F**) surviving cell numbers in KELLY cells treated with ethanol (etoh) or 5 µM oxidovanadium complexes with and without 10 µM BSO, with statistical analysis carried out with ANOVA and Bonferroni post hoc, *ns* not significant, ***p < 0.001 (n = 3). (**G**) effect of oxidovanadium complexes on cellular glutathione levels. IMR32 and KELLY cells were untreated (UT) or treated with BSO, BMOV and AL1–4 at the concentrations shown. After 24 h, the relative, reduced glutathione levels were assessed using MCB. ANOVA with Dunnett post hoc for each cell line using UT. ***p < 0.001 (n = 3).

Our previous work shows that glutathione (GSH) suppression by buthionine sulfoximine (BSO) enhances BMOV-induced cytotoxicity in neuroblastoma cells, possibly by shifting vanadium to its most oxidised state^35^. This previous study demonstrated that several neuroblastoma cell lines, including IMR32, tolerate up to 200 µM BSO with little deleterious effect. Here we show that 10 µM BSO suppresses GSH by 65% (IMR32) and 75% (KELLY) (Fig. 2G), yet again BSO has no significant effect on cell survival when used alone (Supplementary Fig. S3). To assess whether BSO enhanced the cytotoxicity of AL1–4, KELLY cells were treated with both AL1–4 and BSO (Fig. 2A–E). The BSO doubled the cytotoxicity of 5 µM AL1, AL2 and AL4, to a related degree as seen with BMOV (Fig. 1F). With AL3 the cell survival was already low and BSO suppressed this further but not to statistical significance. The AL1–4 complexes therefore show enhanced cytotoxicity during glutathione suppression, confirming that they behave in a similar manner to BMOV^35^.

### Glutathione depletion does not correlate with AL1–4 cytotoxicity

One property of oxidovanadium complexes is their potential to generate reactive oxygen species (ROS), which in turn can induce non-specific toxicity^13,41^. We have shown previously that BMOV appears not to kill cells using this mechanism^35^. Total cytoplasmic glutathione levels, as the cell’s major antioxidant defence, can be used as an approximate reflection of oxidative stress, since oxidised glutathione is exported from cells. Total intracellular glutathione was measured using monochlorobimane (MCB) following 24 h of oxidovanadium compound treatment^42^. As expected, glutathione was significantly reduced by BSO in IMR32 and KELLY, but there was no significant change in glutathione after treatment with either BMOV or AL1–4 (Fig. 2G). These data suggest that elevated ROS production is unlikely to be a major factor underlying the cytotoxicity of these complexes.

### Induction of cell differentiation

**Figure 3:**
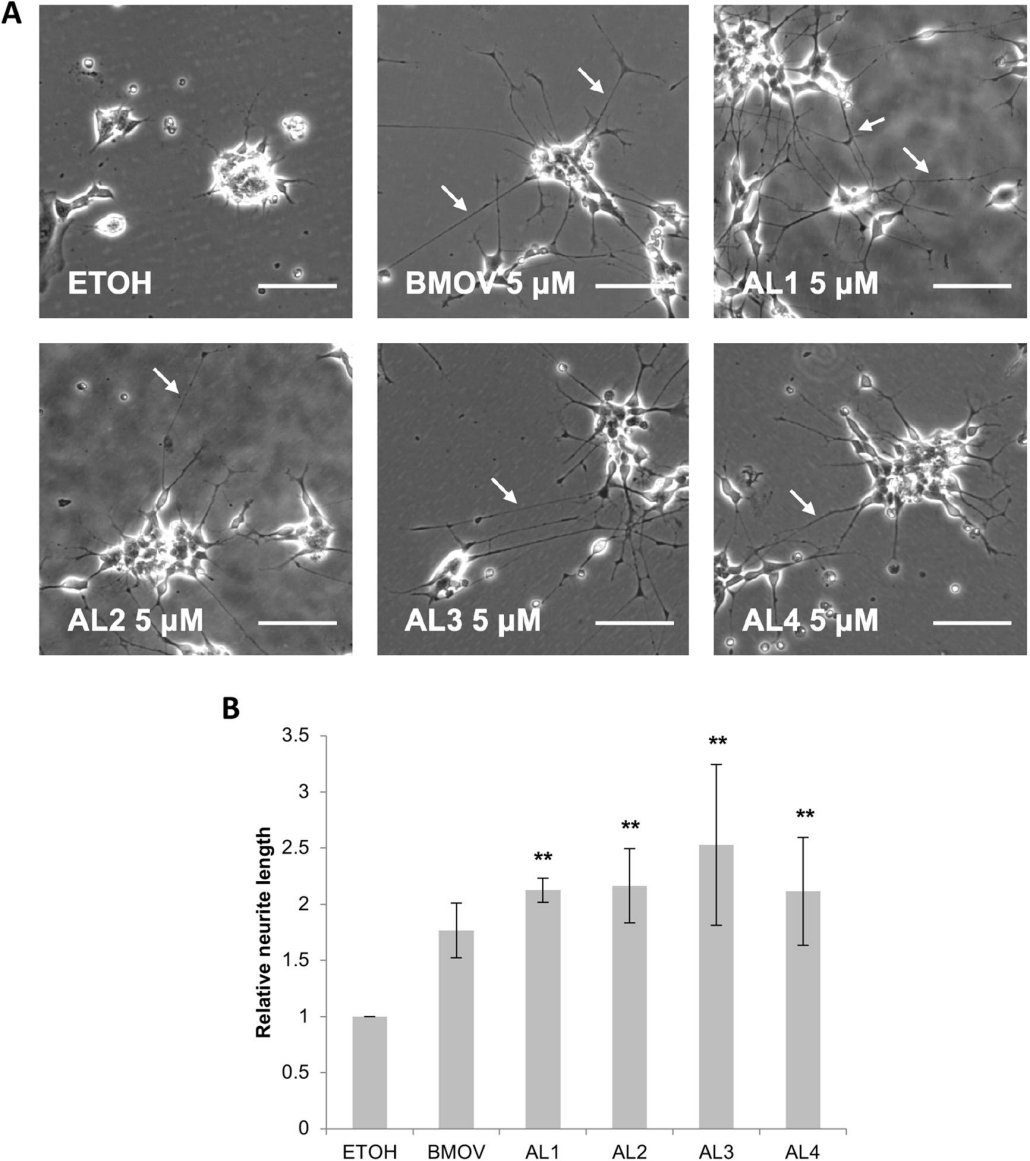
Hydrophobic oxidovanadium complexes induce neurite outgrowth. (**A**) SK-N-SH cells were treated with 5 µM oxidovanadium complexes for 5 days, scale bars = 100 µm. Neurites are indicated with white arrows. (**B**) relative neurite length measurements after 5 days. **p < 0.01 (n = 3) compared to ethanol (ETOH).

Oxidovanadium complexes such as BMOV can elicit the positive response of neuronal differentiation in some neuroblastoma cell lines such as SK-N-SH^36^. SK-N-SH were therefore treated for 5 days with BMOV and AL1–4, and neurite lengths were measured as a parameter of differentiation (Fig. 3). All complexes induced differentiation. Although the data scatter precluded statistical significance, the complexes were all more effective on average than BMOV, with AL3 generating the longest neurites. These hydrophobic oxidovanadium complexes can all therefore trigger morphological, neural differentiation in SK-N-SH cells, rather than cytotoxicity, in a similar manner to BMOV.

### AKT and ERK activation by AL1–4

**Figure 4:**
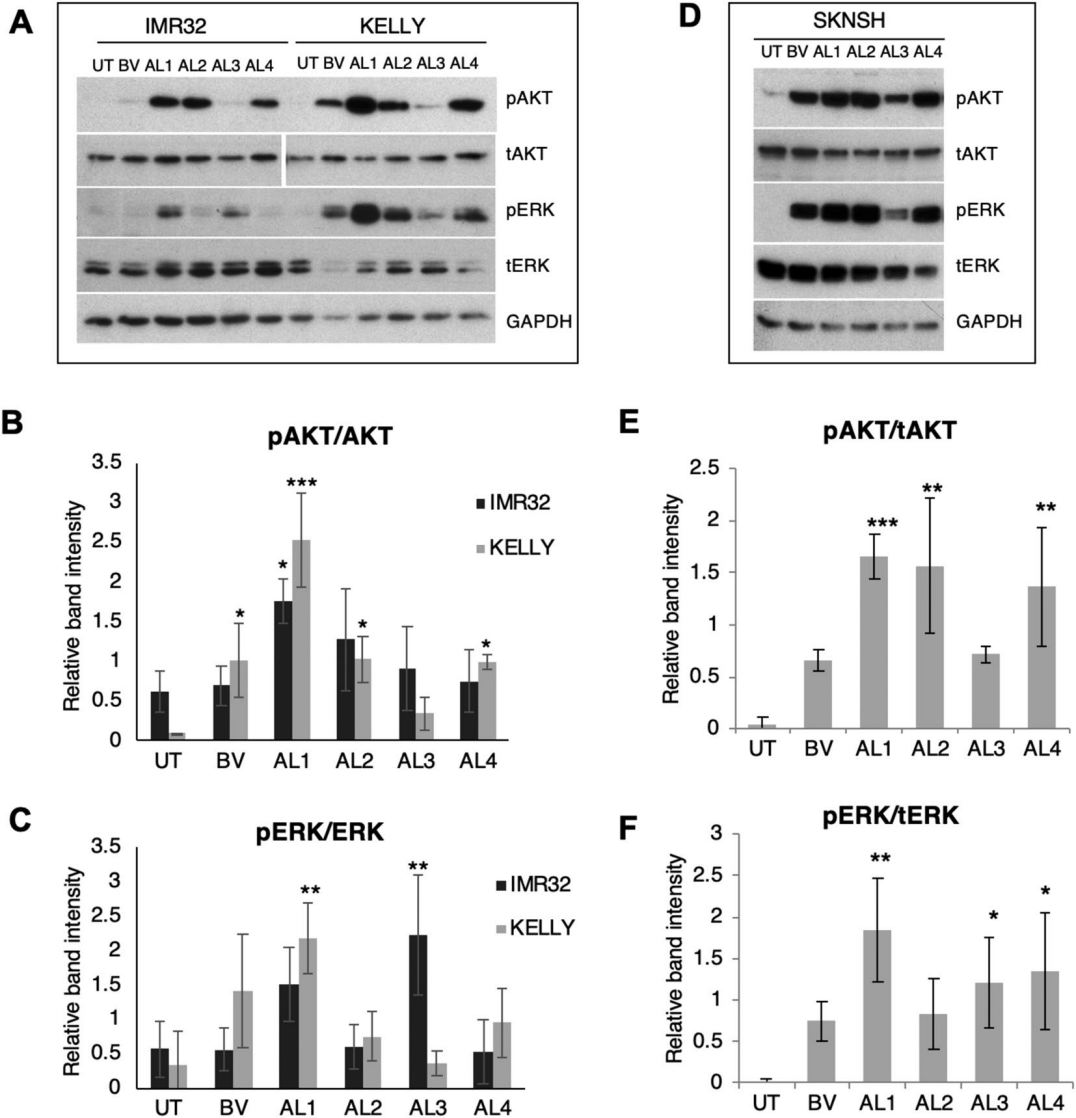
Induction of AKT and ERK signalling by hydrophobic oxidovanadium complexes. (**A**) IMR32 and KELLY cells were treated with 10 µM BMOV and AL1,2,4, and 5 µM AL3 for 48 h. Representative immunoblots for phosphorylated and total AKT and ERK are shown; the AKT bands are taken from different exposures of the same gel. (**B**,**C**) Immunoblots were quantified and relative band intensities for phosphorylated proteins were normalised to total AKT or ERK proteins (n = 3). (**D**) SK-N-SH cells were treated with 10 µM BMOV and AL1,2,4 and 5 µM AL3 for 72 h. Representative immunoblot detecting phosphorylated and total AKT and ERK is shown. (**E**,**F**) immunoblots were quantified and relative band intensities for phosphorylated proteins were normalised to total AKT or ERK proteins. ANOVA with Dunnett post hoc for each cell line using untreated (UT) as the control, *p < 0.05, **p < 0.01, ***p < 0.001 (n = 3). Original gels are shown in Supplementary Figs. S5 and S6.

Increased phosphorylation of AKT and ERK is characteristic of neuroblastoma cells treated with BMOV and vanadate^35,36^. To assess whether AL1–4 similarly enhance these signalling events, IMR32, KELLY and SK-N-SH cells were treated with 10 µM BMOV, AL1, AL2 and AL4, and 5 µM AL3. In IMR32 cells the phosphorylation of AKT was weakest with BMOV, AL3 and AL4, but stronger with AL1 and AL2. In IMR32, phosphorylation of ERK was not significantly enhanced by BMOV, AL2 and AL4, but was enhanced most by AL1 and AL3 (Fig. 4A–C). In KELLY cells, phosphorylation of both AKT and ERK is enhanced strongly and similarly by BMOV, AL2 and AL4, but even more strongly by AL1. In contrast, AL3 only weakly stimulated ERK and AKT phosphorylation (Fig. 4A–C). In SK-N-SH cells, phosphorylation of AKT was strongly enhanced by BMOV and more strongly again by AL1,2,4 (Fig. 4D–F). AL3 was the weakest of the AL complexes in stimulating pAKT. In SK-N-SH, pERK was generated similarly by all complexes, but quantitation showed that on average AL2 was weakest and AL1 strongest. AL1–4 can therefore stimulate pAKT and pERK similarly to, and sometimes more than, BMOV, with compound AL1 stimulating the greatest biochemical changes in all three cell types. Although some of AL3’s reduced signaling in KELLY and SK-N-SH could be explained by the lower dose of 5 µM used, the further qualitative differences seen, for example high stimulation of pERK in IMR32, suggest that AL3 has distinct effector properties. The data also indicate that stimulation of AKT and ERK signaling do not correlate with the relative cytotoxic potential of AL1–4 (Fig. 1). This potentially concurs with our previous conclusions that BMOV does not require AKT or ERK activation for its cytotoxicity^35^.

In summary, the above data show that AL1–4 have broadly similar cellular and biochemical properties compared with BMOV in KELLY, IMR32 and SK-N-SH, while generating more potent effects in some cases.

### AL1–4-induced responses are not driven by their organic ligands

**Figure 5:**
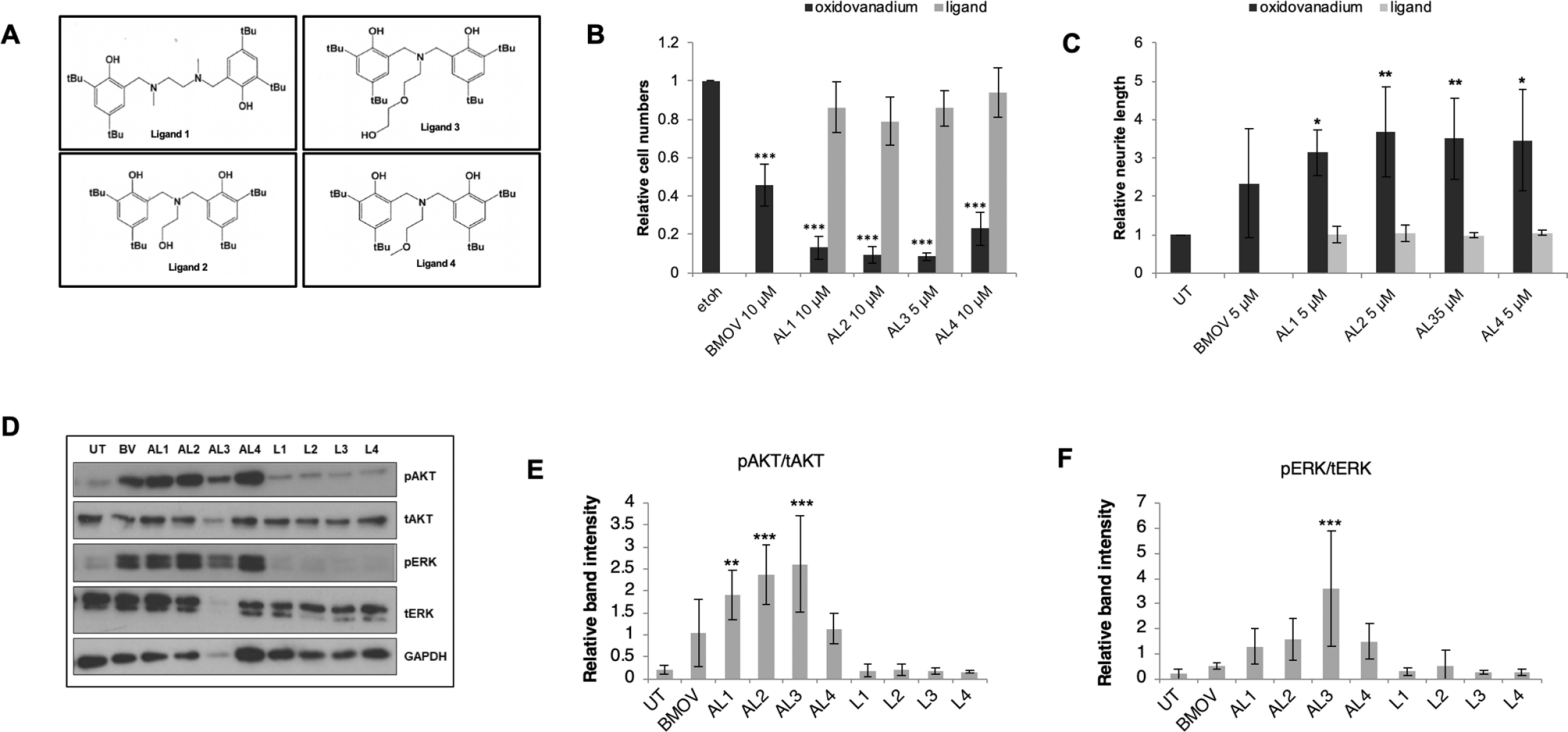
Cellular and biochemical effects of ligands. (**A**) Ligands 1–4 are from the respective hydrophobic oxidovanadium complexes AL1–4. (**B**) IMR32 cells were treated with 10 µM oxidovanadium or ligands only (5 µM for AL3 and L3) for 3 days. Relative cell numbers were assessed using nuclear counting. ANOVA with Dunnet post hoc using untreated (UT) as control, ***p < 0.001 (n = 3). (**C**) SK-N-SH cells were treated with either 5 µM oxidovanadium complexes or respective ligands for 6 days, after which relative neurite lengths were assessed. ANOVA with Dunnet post hoc using UT as the control, *p < 0.05, **p < 0.01 (n = 3). (**D**) representative immunoblot for phosphorylated and total AKT and ERK performed using protein lysates from SK-N-SH cells in (**C**). (**E**,**F**) Immunoblots were quantified from 3 independent experiments and relative band intensities for phosphorylated proteins were normalised to total AKT or ERK. ANOVA with Dunnett post hoc was applied using UT as the control, **p < 0.01, ***p < 0.001. Original gels are shown in Supplementary Fig. S7.

Oxidovanadium complexes with diverse liganding molecules have been used in many cancer models^43,44^. The ligands in some cases have intrinsic anti-cancer properties and the dissociation or speciation of complexes in the gut or circulation is viewed as a pharmacological problem due to off-target toxicities^9,10,22,23,45–47^. In this project we aimed to use the ligands to facilitate oxidovanadium delivery, rather than acting toxically themselves. Since AL1–4 have some quantitative differences in cellular potency and qualitative differences in biochemical responses, they are unlikely to be simply releasing common, dissociated vanadate before entering the cell. It is feasible instead that the ligands may be generating some of these differences. The ligands themselves were therefore investigated for cytotoxic and cell signaling potential (Fig. 5). Free ligands were dissolved in ethanol and cells were treated for 3 days. These free ligands had no observable cytotoxic effects on IMR32 cells (Fig. 5B). SK-N-SH cells treated with AL1–4 generated extended neurites, whereas treatment with the ligands did not (Fig. 5C). Western blotting of SK-N-SH lysates showed that while AL1–4 stimulated phosphorylation of AKT and ERK, the ligands again did not (Fig. 5D–F). These data thus demonstrate that the ligands in AL1–4 do not have cytotoxic, differentiative or AKT/ERK signaling properties in these tumour cells. We have also demonstrated that molybdenum complexes with the same ligand as AL2 are not cytotoxic in IMR32 and do not induce differentiation in SK-N-SH cells (Supplementary Fig. S4).

### Liposomal delivery of AL3

The hydrophobic complexes AL1–4 display similar or stronger activity than BMOV in neuroblastoma cells and may, therefore, have potentially improved anti-cancer activity. However, their strong hydrophobicity would present delivery and bioavailability barriers in vivo. Our second, key aim therefore was to demonstrate that these complexes can be delivered using a method that should in future be compatible with systemic delivery and stability. The approach we have initially tested is that of liposomal encapsulation of AL3, followed by tests of its bioavailability in cultured neuroblastoma tumour cells. AL3 was chosen for this liposomal testing since it was the most hydrophobic and demonstrated the greatest cytotoxicity and differentiative potential.

**Figure 6:**
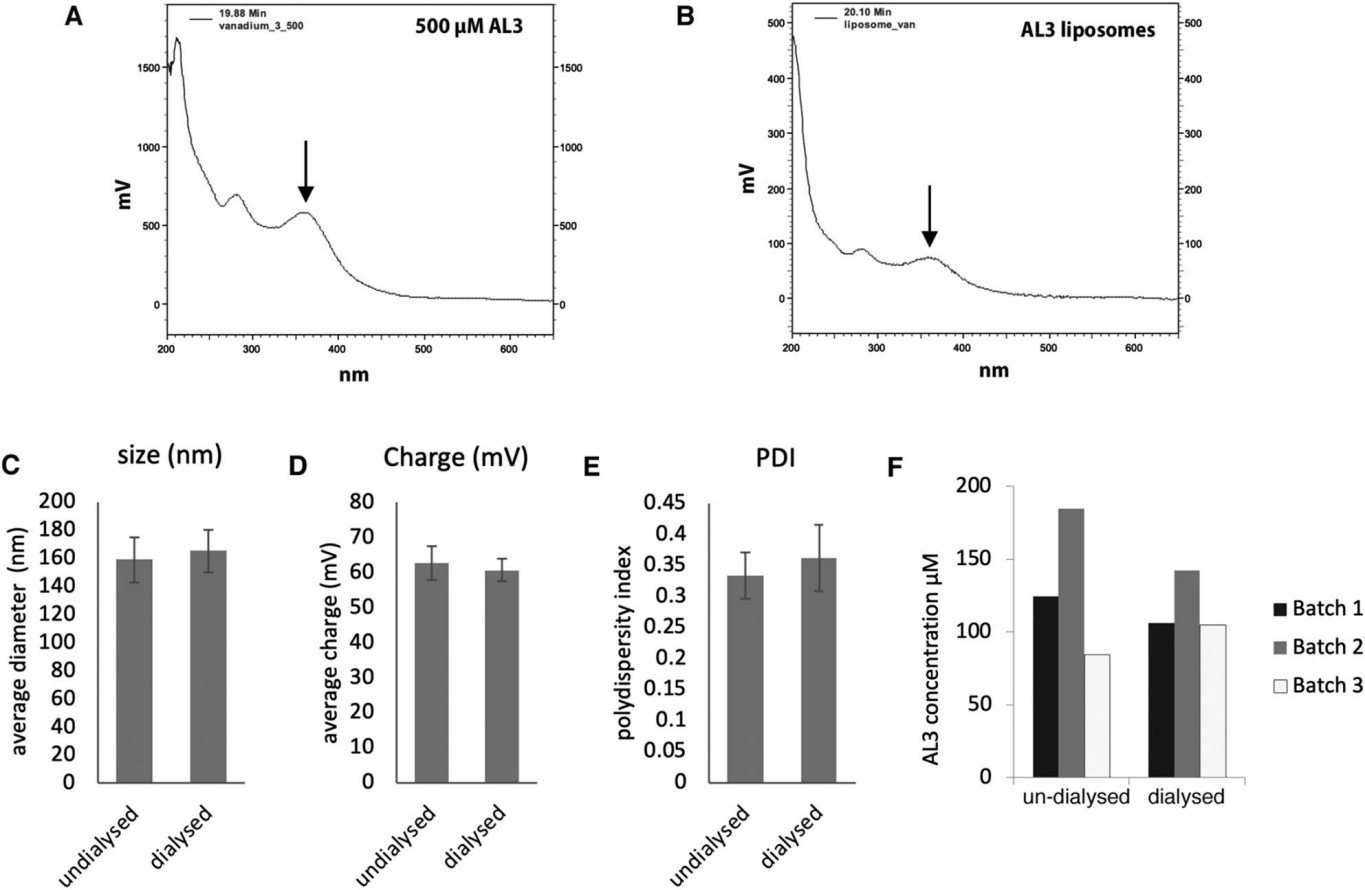
Oxidovanadium complex loading into liposomes. Three batches of AL3 liposomes were synthesised then dialysed over 24 h against water. Reverse-phase HPLC was used to measure AL3 concentration; example UV absorbance traces for ethanol solubilised (**A**) and liposomal (**B**) AL3 are shown. AL3 concentration was assessed using absorbance at 360 nm (black arrows). Average liposome charge (**C**), size (**D**) and polydispersity (**E**) were measured before and after dialysis (n = 3). (**F**) Liposomal AL3 concentration was calculated pre- and post-dialysis in three batches of liposomes.

AL3 was incorporated into liposomes derived from DOPC, a neutral lipid used to promote stable lamellar structures, cholesterol to increase stability of the liposomes, and DOTMA as a cationic lipid to improve uptake in mammalian cells^48^. Liposomes containing AL3 were cationic with an average charge of 64 ± 3 mV, a mean diameter of 159 ± 16 nm and polydispersity index of 0.33 ± 0.04. A reverse-phase HPLC assay was used to measure the concentration of AL3 incorporated into three liposome batches^49^ (Fig. 6A,B). A mean of 131 µM across three liposome batches was calculated, albeit with a broad range, compared with the theoretical maximum loading of 154 µM (Fig. 6F). This showed that, on average, up to 85% of AL3 was incorporated into the lipid compartment of the liposomes. To separate encapsulated from non-encapsulated AL3, liposomes were dialysed within 48 h of synthesis, using dialysis chambers with a molecular weight cut-off of 10 KDa. Size and charge analysis showed no significant changes after dialysis, and AL3 quantitation showed only a small and not statistically significant decrease of AL3 concentration after dialysis (Fig. 6F). This indicates that AL3 can remain stably incorporated in these liposomes for at least 48 h (Fig. 6C,D). In the experiments below, we used the theoretical concentrations of AL3 based on maximal loading, rather than the actual amount loaded. This was necessary since we needed to maintain consistency in, and also to minimise, the quantity of liposomal material we treated the cells with.

### Cytotoxic activity of liposomal AL3

**Figure 7:**
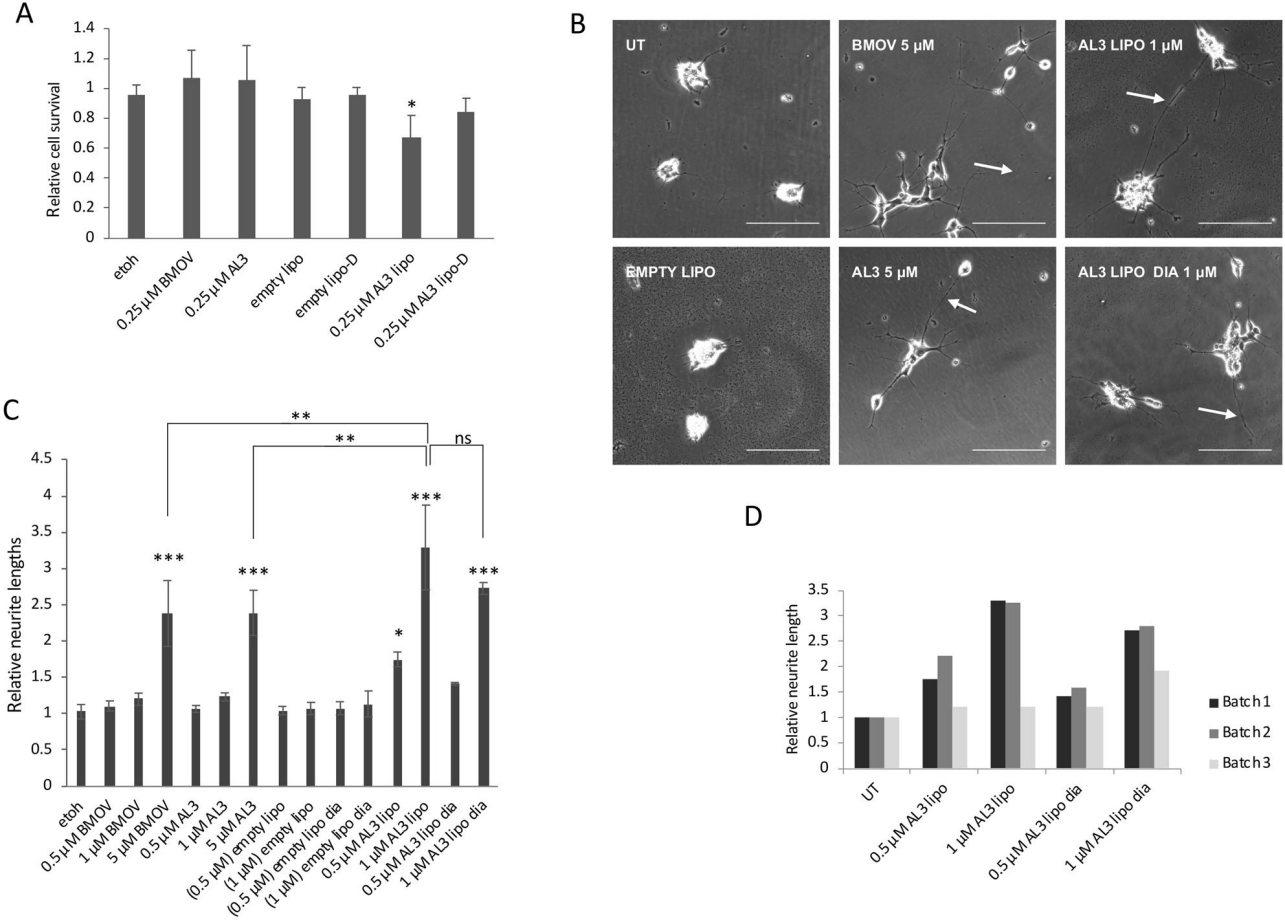
Biological effects of AL3-containing liposomes in neuroblastoma cells. (**A**) IMR32 cells were treated for 6 days with BMOV or AL3 in solution, empty liposomes, or AL3 liposomes (batch 1; both undialysed and dialysed [D]) after which cell viability was assessed by nuclei counting. ANOVA with Dunnet post hoc using ethanol (etoh) as the control, *p = 0.053 (n = 3). (**B**) SK-N-SH cells were treated for 5 days with BMOV or AL3 in solution, undialysed empty liposomes, or batch-1 undialysed (AL3 LIPO) and dialysed (AL3 LIPO DIA) AL3 liposomes. Neurite formation (white arrows) is indicated. (**C**) Neurite lengths were measured after treatments with BMOV, AL3, empty liposomes and AL3 liposomes. Dialysed liposome treatments are indicated as “dia”. Empty liposome dosage in parenthesis matches the lipid content of those used in the equivalent AL3 liposome treatments. ANOVA with Dunnett post hoc using untreated ethanol (etoh) as control **p < 0.01, ***p < 0.001 (n = 3). (**D**) SK-N-SH cells were treated in the same way as in (**B**) and (**C**), using batch 2 and 3 AL3 liposomes, showing batch 1 data again for comparison. Mean average effects on neurite length are shown.

To assess the cellular bioavailability and activity of liposomal AL3, IMR32 cells were treated with liposomes for 6 days at 0.25 µM AL3. Higher concentrations of AL3 could not be used in IMR32 because they become subject to non-specific toxicity from these particular liposomes. Nevertheless, at 0.25 µM the liposomal AL3 generated a 25–30% reduction in cell viability over 6 days (Fig. 7A). This equates approximately to the toxicity seen with the equivalent of 0.8 µM of ethanol-solubilised AL3 over 3 days (Fig. 1B). Although these treatments are for different times, the cells appear to be responding similarly given that the 0.25 µM of ethanol-solubilised AL3 in Fig. 7A causes a small increase in cell numbers that remains comparable to that seen in Fig. 1B.

### Cell differentiation by liposomal AL3

To measure the stimulation of cell differentiation, the neuronal SK-N-SH cells were treated with BMOV or AL3 in solution, with dialysed and non-dialysed liposomal AL3, or with empty liposomes. Average neurite lengths were quantified after 5 days (Fig. 7B). BMOV and ethanol-solubilised AL3 induced increased neurite outgrowth. Neither the empty liposomes, nor BMOV or AL3 in solution at concentrations below 5 µM affected neurite length in SK-N-SH, and they were not toxic. Liposomal AL3, dialysed and non-dialysed, was better tolerated in SK-N-SH than seen with IMR32 above and significantly increased neurite length at 0.5 µM and 1 µM AL3 (Fig. 7C). Similar neurite outgrowth stimulation was obtained with a second liposomal AL3 batch. A third batch (batch3; Fig. 6E) stimulated lesser neurite outgrowth, but had lower undialysed AL3 content (Fig. 7D). At 1 µM, undialysed and dialysed liposomal AL3 increased neurite lengths to a greater or equal extent, respectively, to those seen with 5 µM soluble AL3 (Fig. 7C). There is a small, but not significant, decrease in neurite length relating to dialysis, broadly mirroring the difference in AL3 concentration in these formulations as described Fig. 6E. These data therefore indicate that liposomal AL3 has a fivefold greater differentiation capacity than AL3 delivered in ethanol.

### Biochemical signaling by liposomal AL3

**Figure 8:**
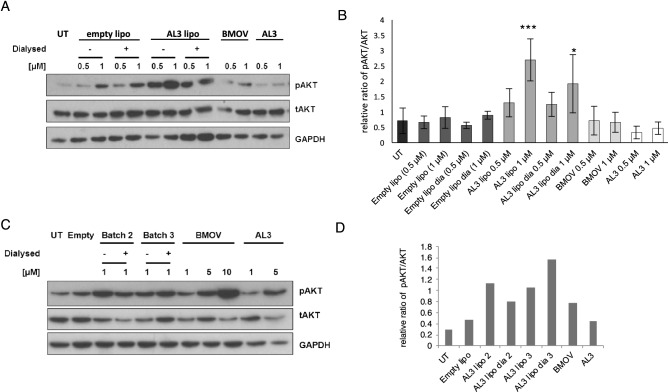
Enhancement of AKT phosphorylation by liposomal AL3. (**A**) SK-N-SH cells were treated with BMOV or AL3 in solution, empty liposomes, or batch-1 AL3 liposomes (both dialysed and dialysed) for 3 days. An example immunoblot is shown showing pAKT stimulation levels; this is quantified in (**B**) along with two further datasets (n = 3). ANOVA was performed with Dunnett post hoc using untreated (UT) as the control, *p < 0.05, ***p < 0.001. (**C**) SK-N-SH were treated in the same way using batch 2 and 3 of the AL3 liposomes and the immunoblotting is shown; this blot is quantified in (**D**). Original gels are shown in Supplementary Figs. S8 and S9).

To test whether liposomal AL3 can trigger AKT signalling, SK-N-SH cells were treated for 3 days with BMOV or AL3 in solution, and with dialysed and non-dialysed AL3 liposomes (Fig. 8). Since SK-N-SH could tolerate higher levels of liposome treatment than IMR32, we used up to 1 µM liposomal AL3 and comparable empty liposome quantities. Significant increases in phosphorylation of AKT were observed in cells treated with 1 µM liposomal AL3, both in dialysed and non-dialysed forms. Similar to the differentiation data, liposomal AL3 was more effective at stimulating signaling compared to ethanol-solubilised AL3. These data indicate that AL3 can be successfully packaged into liposomes and that liposomal delivery improves the ability of AL3 to counter the cancer cell phenotype compared with ethanol-solubilised AL3 in cultured neuroblastoma cells.

## Discussion

The four oxidovanadium complexes described in this study are based on amine bisphenol, aminoalcohol bisphenol or salan ligands. They are highly hydrophobic and are able to effect either cytotoxicity or differentiation in several neuroblastoma tumour-derived cell lines. They are also as effective, or more effective, than the previously documented actions of a water soluble oxidovanadium complex BMOV in these cells^35,36^. The current study is also significant in that it demonstrates for the first time that with liposomal technology such oxidovanadium complexes can be made bioavailable and show increased anti-neuroblastoma cell activity.

The oxidovanadium complexes AL1–4 behave similarly to BMOV in that their effects are enhanced by blockade of GSH production. This may relate to the fact that tumour cells, including MYCN-amplified neuroblastoma cells, harbour elevated levels of GSH to compensate for, and defend against, higher ROS production^50^. A minority of neuroblastoma tumour cell lines are also directly sensitive to GSH depletion itself^51^. Oxidovanadium is sensitive to the REDOX state of cells and our complexes become more toxic when GSH is depleted. As previously hypothesised with BMOV^35^, the more oxidising conditions should generate higher cellular levels of the vanadium(V) ion, which is a more effective phosphatase inhibitor^35^, and consequently higher cytotoxicity. Oxidovanadium complexes can have lower cytotoxicity in non-tumour cells compared to tumour cells^35,52,53^ and it will be of interest to examine if this relates to these cells’ more reducing environment.

It is also of interest that low doses of these complexes increase cell numbers in KELLY, and to a lesser degree in IMR32, before switching to cytotoxicity at higher doses. This has been described before in MC3T3E1 osteoblast-like cells^54^ but is still not understood. It may relate to the differential sensitivity of the multiple PTP targets of oxidovanadium. Additionally, oxidovanadium has diverse biochemical effects in cells and this may be concentration-dependent and also differentially controlled by cellular REDOX state. Nevertheless, when used in vivo, oxidovanadium compounds including BMOV are reasonably well tolerated while having chemopreventive and anti-tumour activities^2^. This indicates that a therapeutic window may be possible with such compounds.

These four oxidovanadium complexes have distinct organic ligands and their relative potencies were quantitatively and qualitatively distinct. AL3 induced strong phosphorylation of ERK in IMR32 but not KELLY cells, despite AL3 being used at a lower dose than the other complexes, while both cell lines have a strong cytotoxic response to this compound. AL1 drove the strongest phosphorylation of AKT and ERK, but was less toxic than AL3 and no more cytotoxic than AL2 or AL4. Although we have shown previously that ERK and AKT activation by BMOV are not needed for its cytotoxicity^35^, this qualitative uncoupling of the morphological and biochemical responses to AL1–4 was not foreseen and may again indicate the lack of necessity for ERK or AKT activation for neuroblastoma cell death or differentiation.

In seeking to understand the basis for the differences in their actions, it was shown that the complexes appeared not to differ significantly in terms of glutathione depletion, and there were no correlations between glutathione levels and cytotoxic capacity. Thus, ROS production through interconversion between the vanadyl (IV) and vanadate (V) redox states^13^ may not be a significant cytotoxic factor here. The qualitative distinctions between AL1–4 also suggest that these complexes are not simply speciating outside cells and acting as vehicles for common, bioactive vanadate. Since AL1–4 have distinct ligands, it is possible that the ligands are generating the complexes’ quantitative and qualitative differences. In less stable oxidovanadium complexes, speciation can release ligands that are themselves toxic^23,24,45^. However, our data indicate that free ligands from AL1–4 have no recognisable biological activity. It is possible that the ligands alone do not enter cells efficiently, but can be carried in by the intact complexes, where they then dissociate and have access to novel targets. This is unlikely in our view, but cannot be excluded currently.

Why is AL3 the most cytotoxic in KELLY and IMR32 cells and is the strongest differentiating agent in SK-N-SH? Although the complex may simply enter cells more efficiently than the others, its unique AKT/ERK signalling pattern would not be consistent with this scenario. It will be of interest in future to examine the relative efficacies of the other compounds when delivered in liposomes, as liposomes may provide more uniform cellular delivery. AL3’s higher activity might instead be explained by it having access to specific, intracellular compartments, providing selective access to differentially localised PTPs^55–57^. For example, a distinct oxidovanadium compound, decavanadate, has been reported to induce mitochondrial membrane depolarisation by localising selectively to mitochondria^58^. Furthermore, although it is likely that AL1–4 speciate inside cells, releasing vanadate, they may also have some differential abilities to inhibit PTPs as intact complexes. For example, the cis-aquo form of BMOV can fit favourably inside the active site of PTP-1B, at least through structural modelling^59^. However, AL1–4 have additional, bulky tert-butyl groups on their phenolic rings and these are likely to preclude access to the PTP active sites. Intact AL1–4 could even have other non-PTP targets in cells and further studies are therefore required in order to explain the cellular and biochemical responses to AL1–4.

An iso-propoxide analogue of AL1, L^1^VO(OiPr), with the same tert-butyl-substituted salan ligand, has been described as being non-cytotoxic in HT29 colorectal and OVCAR-1 ovarian cancer cells^40^. It was suggested, but not demonstrated, that this was due to either its stronger hydrophobicity, poor membrane accessibility or inability to associate with intracellular targets^40^. In contrast, AL1 is evidently cytotoxic in neuroblastoma cells and can also induce neuronal differentiation. We currently do not know why it shows this activity in neuroblastoma cells compared to HT29, but the somewhat less hydrolytically stable methoxide versus more stable iso-propoxide modification, may contribute. The team that generated L^7^VO(OiPr) later developed hydrophobic and highly stable diamino tris(phenolato) liganded oxidovanadium complexes and showed their high cytotoxicity in culture and ability to suppresses HT-29 tumours in vivo^29^. Our data with complexes AL2–4 therefore concur with Reytman’s study, supporting the improved therapeutic opportunities that could be offered through use of such stable, hydrophobic oxidovanadium complexes.

Although hydrophobic complexes AL1–4 may offer improved anti-cancer opportunities, such complexes would present challenges as therapeutics due to their extreme insolubility. Our key aim here was to bring nanotechnology to bear on this problem and demonstrate cellular delivery of the complexes using liposomes. In this study we used cationic liposomes, having previously found that related cationic formulations are effective in drug delivery in culture and in vivo and are poor activators of the complement system^60,61^. Our previous work on RAMBA chemicals that are similarly hydrophobic to the present oxidovanadium complexes, also showed very effective payload delivery by liposomes in under 24 h, and others describe this occurring in under 6 h^48,62,63^. We demonstrated here in this proof-of-principle study that the liposomal delivery approach for oxidovanadium complexes works well under cell culture conditions. This is as far as we know the first demonstration of liposomal-dependent bioavailability of hydrophobic oxidovanadium complexes, with apparent enhancement of cytotoxic and differentiative potential. Interestingly, as we completed this work, a biophysical study documented the successful loading of liposomes with a curcumin complex of oxidovanadium. It will be interesting to see in future whether these are also effective at delivering curcumin to cancer cells^64^.

Liposomal formulations are used for delivery of a range of anti-cancer drugs in human trials and some are now in the clinic^65^. This has also been recently reviewed for neuroblastoma^66^. Liposomes can penetrate some solid tumours by ‘passive targeting’ through the enhanced permeability and retention (EPR) effect^67^. Although this appears feasible in neuroblastoma xenograft models^68,69^, it is unclear whether EPR is relevant here clinically. The delivery of oxidovanadium complexes in vivo for neuroblastoma would likely need more advanced liposomal technology, such as tumour targeting with RGD-based peptides^60,69^, or antibodies specific for disialoganglioside 2 (GD2), a key antigen on neuroblastoma cells^70,71^. PEGylated lipids can also be employed. PEGylation often reduces transfection efficiency in cultured cells^72,73^, whereas in vivo they increase liposomal longevity in the bloodstream and reduce immunological responses and drug toxicities^73–75^.

The potential benefits of a liposome-based route of oxidovanadium complex delivery are several-fold. First, the hydrophobic complexes would likely partition strongly in the liposomes rather than the aqueous circulation, discouraging speciation and encouraging stability. We would in future nevertheless need to examine this liposomal stability under high serum conditions in vivo, even though we have shown that similar liposomal systems can deliver payloads quite efficiently either systemically or locally^61^. Second, if the more targeted and selective delivery of oxidovanadium to tumour tissues is attainable, this would significantly reduce the concerns of off-target toxicities that have beset other oxidovanadium complexes in chronic disease treatment models. Finally, since liposomal AL3 induced several-fold stronger cell differentiation and AKT activation compared to ethanol delivery, this liposomal approach may generate a better therapeutic index at lower oxidovanadium doses in vivo, again offsetting concerns with off-target toxicities. The liposomal packaging of oxidovanadium complexes is therefore demonstrated to be a viable approach and should be further tested now in pre-clinical models. Ultimately it would be envisioned for delivery as an intravenous therapeutic, in common with current liposomal formulations^65^.

In conclusion, we have provided evidence of good bioactivity of stable, hydrophobic oxidovanadium complexes in neuroblastoma cells. Moreover, in the first proof-of-concept, we can now deliver such complexes using liposomal nanotechnology, opening up new opportunities for demonstrating the anti-cancer potential of oxidovanadium complexes in pre-clinical cancer models. This would not only be of relevance to neuroblastoma, but also other oxidovanadium-sensitive cancers. If successful pre-clinically, this would strengthen the prospects of generating liposomal formulations for the safe application of oxidovanadium complexes in humans.

## Materials and methods

### Cell culture

Neuroblastoma cell lines used were as follows: KELLY/N206 cells were kindly provided by Prof. Frank Speleman, University of Ghent; SK-N-SH and IMR32 cells were from ATCC. All cells were STR validated at source or in our laboratory. All cells were cultured in RPMI 1640 + GlutaMAX (Gibco), 10% foetal bovine serum (FBS) (Gibco), 100 U/ml penicillin and 100 µg/ml streptomycin (P/S; Gibco), except for SK-N-SH, which were cultured in MEM (Gibco), 10% FBS, 1% P/S, 2 mM l-glutamine (Invitrogen, California, USA). Cells were maintained at 37 °C in 5% CO_2_ and high humidity.

### Chemicals

All chemicals were purchased from Sigma-Aldrich, UK unless otherwise stated. BMOV was from ORGANICA Feinchemie GmbH Wolfen, Germany. Complexes AL1–4 and their ligands were synthesised as described previously^37–39^. The solubility of these complexes in water is negligible as measured by spectroscopic (UV, 51 V NMR) methods. For treatment of cells, the compounds AL1–4 were dissolved in ethanol and used at a final solvent concentration of 1% on cells, controlled for by using solvent-only treatments.

### Monochlorobimane assay

Cells were seeded into black-walled 96-well plates (Corning Costar TC-Treated 96-well assay plates) and treated with chemicals the following day in triplicate. At the end of the assay, media was removed and replaced with 100 µl of 50 µM monochlorobimane (MCB) diluted in PBS. Fluorescence was read immediately and after 75 min using a FLUOstar OPTIMA plate reader (BMG LabTech, UK) (absorbance 360 nm; emission 460 nm).

### Neurite length assay

Five thousand SK-N-SH cells were seeded per well in 12-well plates and chemically treated the next day. After treatment, phase-contrast images were recorded (ten fields of view per well) with an Olympus IX71 inverted microscope and a Hamamatsu Orca R2 monochrome camera and randomised for blind analysis. The length of the neurites in each image was measured using the NeuroJ plugin on ImageJ^76^. Only neurites longer than the cell body were measured and an average neurite length per field was calculated.

### Cell viability assays

The remaining cells after cell viability assays were counted using nuclear labelling with Hoechst 33342. Half of the cell media was removed and replaced with fresh media containing 2 μg/ml Hoechst 33342 (Cayman Chemical, Michigan, USA; final concentration 1 μg/ml). Plates were incubated for at least 30 min, before fluorescence imaging. The ImageJ ‘find maxima’ tool was used with a tolerance of 2 to count nuclei in each image. Low-power images were taken in the centre of each well and the average nuclear count calculated across the triplicate wells. For crystal violet assays, cells were seeded in triplicates and at the end point of experiments the media was removed and cells were fixed in 4% paraformaldehyde (PFA) at room temperature for 10 min. Cells were stained with crystal violet (1% crystal violet; 15% methanol) for 1 h at room temperature, washed in distilled water, and air dried overnight. Crystal violet stain was solubilised in 20% acetic acid (v/v) by shaking at room temperature for several hours, and absorbance at 595 nm was measured using a FLUOstar OPTIMA plate reader (BMG LabTech, UK). Resazurin (Sigma-Aldrich) was used according to the manufacturer’s protocol.

### Immunoblotting

Proteins in cell lysates were prepared in 50 mM Tris-base, pH 7.6; 150 mM NaCl; 1% Triton X-100; 0.02% sodium azide. 1 mM protease inhibitor cocktail (Roche, Switzerland) was added along with 1 mM sodium orthovanadate and 25 mM sodium fluoride. Protein concentrations were calculated using the Bradford reagent (Bio-Rad, California, USA) as per manufacturer’s instructions. Lysates were mixed with 4 × sample loading buffer (25 mM Tris–HCl, pH 6.8; 60% glycerol (v/v); 8% SDS (w/v); 10% (v/v) β mercaptoethanol), heated to 100 °C for 5 min, then separated using sodium dodecyl sulfate–polyacrylamide gel electrophoresis in a Mini-PROTEAN Tetra assembly (Bio-Rad). Precision Plus Protein Standards (Bio-Rad) were used. After transfer to polyvinyldifluoride (Immobilon—Merck Millipore, Massachusetts, USA) using a wet transfer in a Bio-Rad Mini Trans-Blot tank, membranes were blocked for 1 h in 10% (w/v) non-fat milk powder (Marvel) dissolved in 50 mM Tris-base, pH 7.4, 150 mM NaCl, 0.1% (v/v) Tween-20 (TBST). Primary antibodies were diluted in blocking buffer with 0.05% (w/v) sodium azide and membranes were incubated overnight at 4 °C followed by washing and incubation for 1 hr with Horseradish peroxidase (HRP)-conjugated secondary antibodies. After washing, HRP was detected using Pierce ECL Plus western blotting substrate (Thermo Fisher Scientific, Massachusetts, USA) and X-ray film (Amersham Hyperfilm, GE Healthcare). Filters were stripped for re-probing using 0.2 M NaOH (20 min at 37 °C followed by 20 min at room temperature). X-ray film bands were quantified using ImageJ. All antibodies were purchased from Cell Signaling Technologies, UK: GAPDH (CST2118), phospo-AKT (CST4060), AKT (CST9272), phosphor-ERK (CST9106), ERK (CST9102).

### Liposomes

Liposomes were synthesised using the thin film hydration method. Compound AL3 was dissolved in a molar ratio of 50:50 methanol:chloroform solution at 5 µg/ml, and stock solutions of lipids DOTMA, DOPC (both from Avanti polar lipids, Alabaster, AL, USA) and cholesterol (Sigma-Aldrich, Dorset, UK) were each prepared at 10 mg/ml in chloroform. DOTMA, DOPC, cholesterol and AL3 were mixed in a round-bottomed flask to a final volume of 500 µl at a molar ratio of 35:35:20:10 (37.5:37.5:25 for empty liposomes). Rotary evaporation (vacuum pump V-700, Rotavapor R-3—Buchi, Flawil, Switzerland) at room temperature for 30 min was followed by rehydration in 1 ml nuclease free water and rotating overnight, followed by sonication for 30–40 min (XB3 Ultrasonic Bath). Size/charge analysis was performed using the Zetasizer Nano ZS system (Malvern Panalytical, UK). The liposomal concentration was 1 mg/ml, giving a final concentration of 0.54 mM for DOTMA and DOPC, 0.31 mM cholesterol and 0.15 mM AL3.

### Liposome dialysis

Five hundred microlitres of liposomes were dialysed within 48 h of liposome synthesis using Slide-A-Lyzer MINI dialysis devices with a molecular weight cut-off of 10 KDa (Thermo Fisher Scientific, Massachusetts, USA). Dialysis was against 13 ml distilled water at 4 °C, shaking for 24 h. The dialysis buffer was replaced twice with fresh sterile water after 4 and 18 h.

### High performance liquid chromatography (HPLC)

Reverse-phase HPLC was used to measure the concentration of compound AL3 within liposomal formulations. 50 µl samples were separated using the JASCO Automated HPLC System. A Hypersil MOS-2 (C8) reverse-phase HPLC column (Thermo Fisher Scientific, Massachusetts, USA—30,305–254,630) was used with 5 µm particle size, 250 mm column length and 4.6 mm internal column diameter. Mobile phase A was water and mobile phase B was 100% acetonitrile. The optimal absorption measurement for AL3 was found to be 360 nm and from this a standard curve was generated using ethanol-solubilised AL3. Data was collected using the EZChrom Elite V 3.17 software.

### Statistics

Data were analysed using analysis of variance (ANOVA). For ANOVA, post hoc testing was performed using the Dunnett test when all experimental conditions were compared to the same control, or Bonferroni when multiple comparisons were needed. Statistical tests were performed using SPSS (IBM SPSS Statistics 25) and significant changes (*p < 0.05, **p < 0.01, ***p < 0.001) are indicated throughout.

## Supplementary information

Supplementary Information.
