# Supplementary material for: The liposomal delivery of hydrophobic oxidovanadium complexes imparts highly effective cytotoxicity and differentiating capacity in neuroblastoma tumour cells
Source: Sci Rep. 2020 Oct 7;10:16660. doi: 10.1038/s41598-020-73539-6 (PMC7542164; doi:10.1038/s41598-020-73539-6)
Supplement: Supplementary file 1 — Supplementary Information. [file 41598_2020_73539_MOESM1_ESM.pdf]

The liposomal delivery of hydrophobic oxidovanadium complexes imparts highly effective cytotoxicity and differentiating capacity in neuroblastoma tumour cells

Elsa Irving, Aristides D Tagalakis[1], Ruhina Maeshima, Stephen L. Hart, Simon Eaton, Ari Lehtonen[2], and Andrew W. Stoker*

Great Ormond Street Institute of Child Health,

University College London, 30 Guilford Street, London, WC1N 1EH

*Corresponding author

a.stoker@ucl.ac.uk

tel:02079052244

[1] Current address: Department of Biology, Edge Hill University,

Ormskirk, L39 4QP, UK

[2] Department of Chemistry, University of Turku, FI-20014 Turun yliopisto, Finland

SUPPLEMENTARY FIGURES

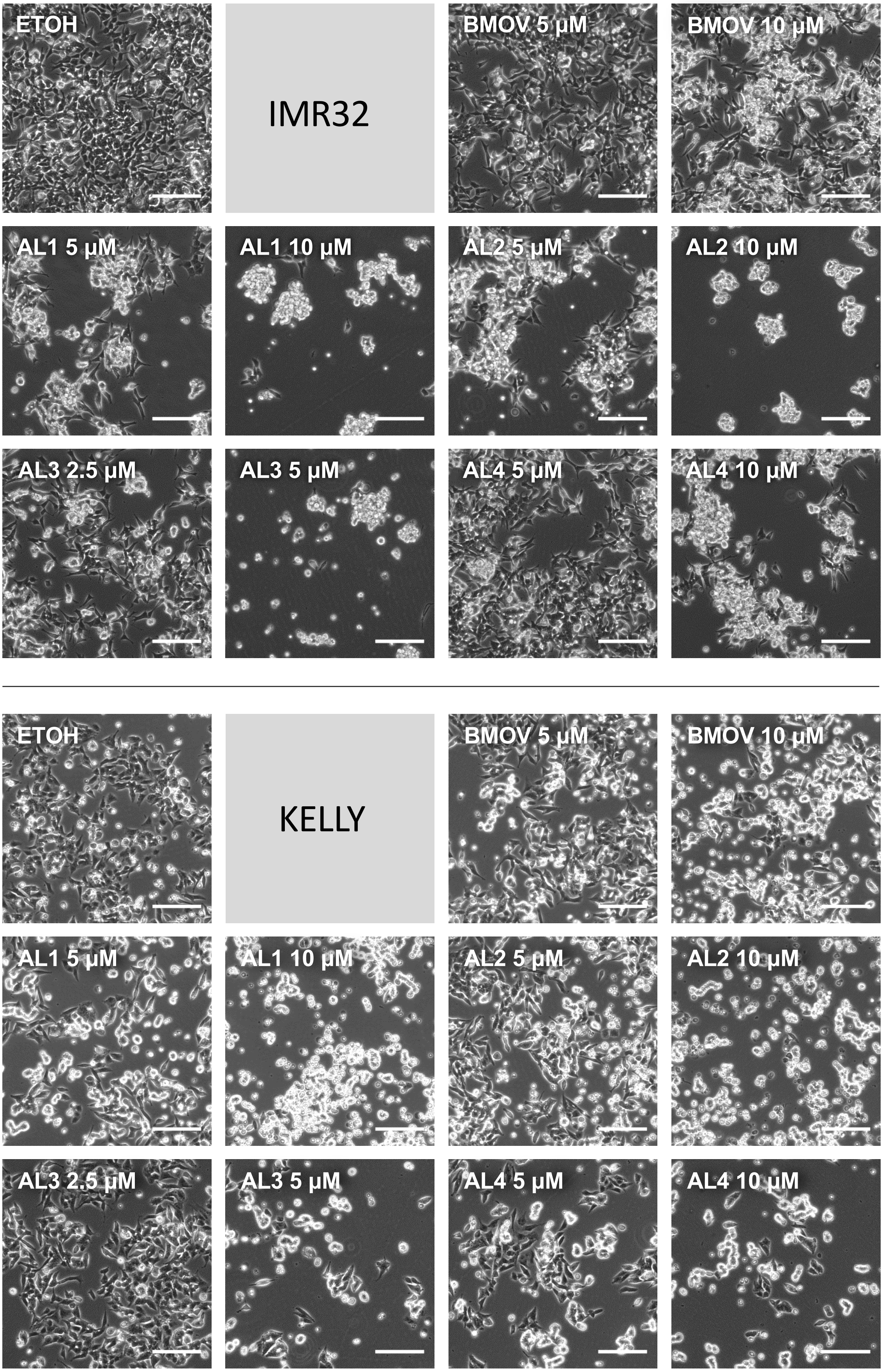

Supplementary Figure S1. Hydrophobic oxidovanadium complexes induce cytotoxicity in neuroblastoma cells. IMR32 cells (top panels) and KELLY cells (bottom panels) were treated with BMOV and oxidovanadium complexes AL1, AL2 and AL4 at 5 µM and 10 µM, or with AL3 at 2.5 µM and 5 µM. Cells were imaged after 3 days. Scale bars = 150 µm.

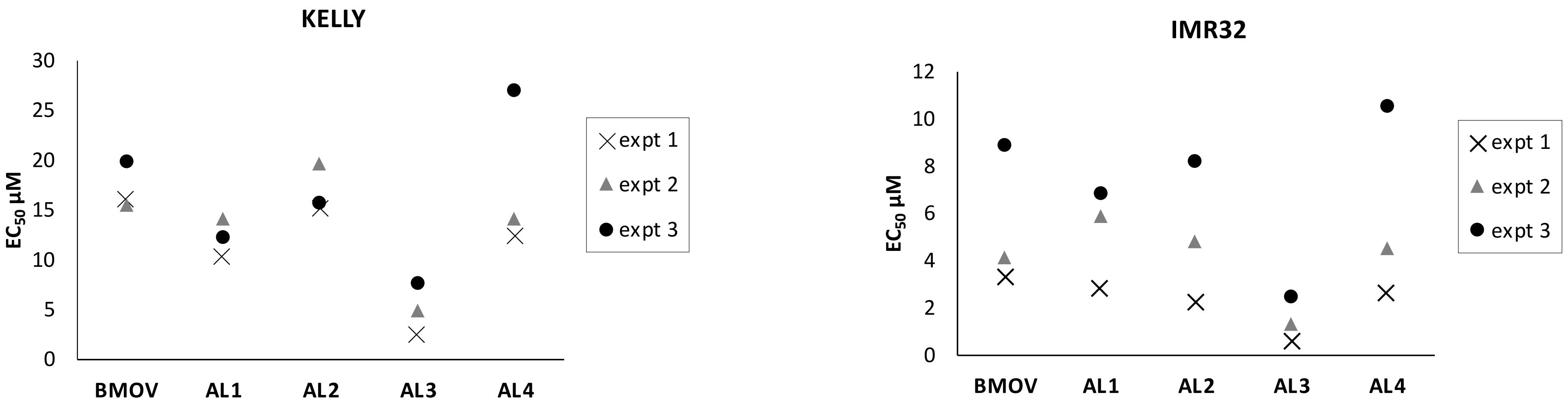

Supplementary Figure S2. EC_{50} data from three treatments of KELLY and IMR32 cells with BMOV, AL1, AL2, AL3 and AL4 as describe din Figure 1C. Note that all KELLY values are only approximations due to having to extrapolate line graphs to reach the 50% cell survival point on the Y-axis (see line charts in Figure 1C).

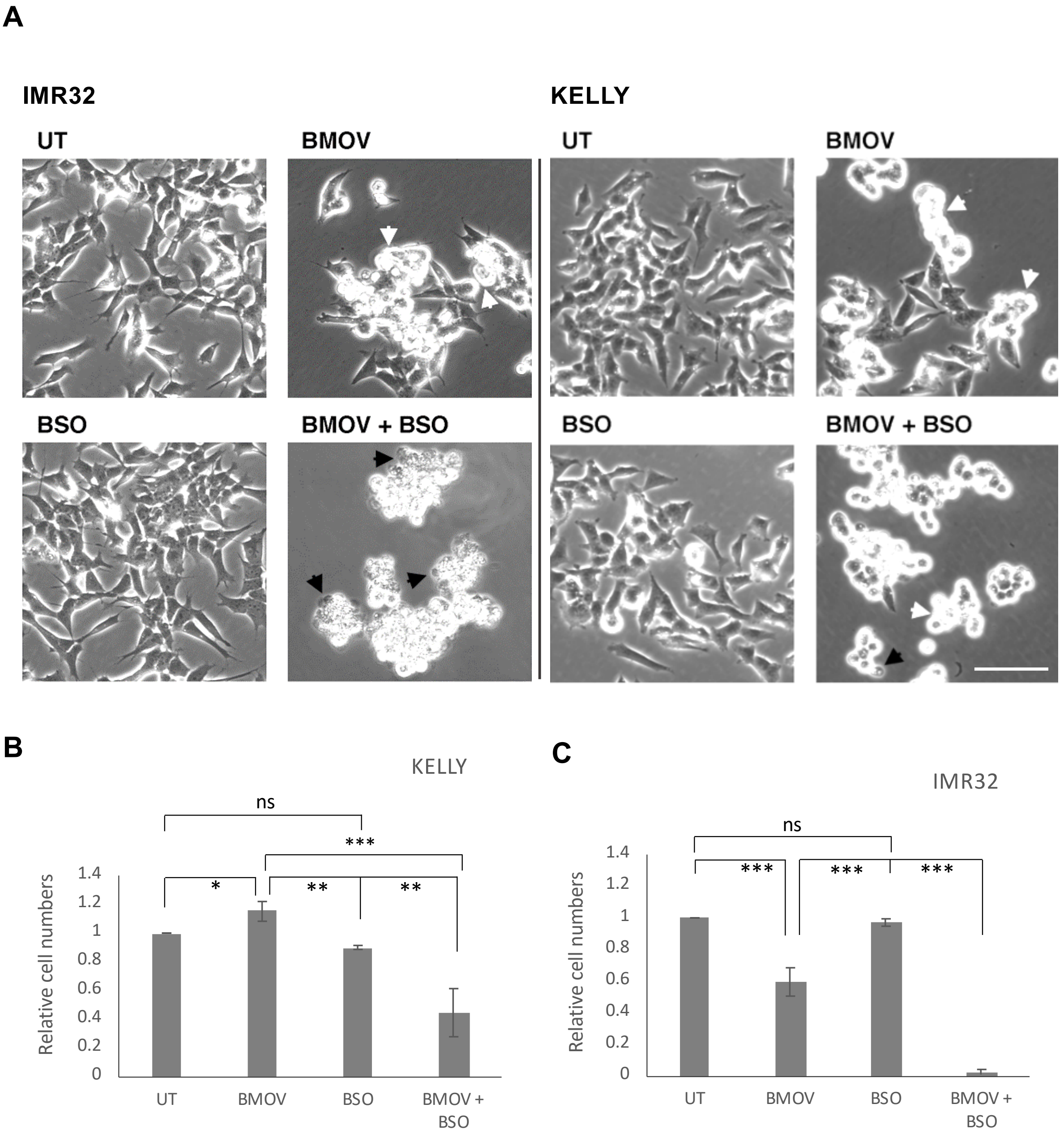

Supplementary Figure S3. Assessing the cytotoxicity of BMOV and BSO in IMR32 and KELLY cells. Cells in 12-well plates were left untreated (UT) or treated for 3 days with 10µM BMOV, 10µM BSO or the combination, then photographed. White arrowheads indicate rounded, refractile and live cells; black arrowheads indicate dead cell bodies. Scale bar = 150µm. Parallel, 96-well survival assays were run for 3 days and processed with resazurin for KELLY (B) and IMR32 (C). Anova analyses were performed, * $P<0.05$, ** $P<0.01$, *** $P<0.001$, ns - not significant, n=3.

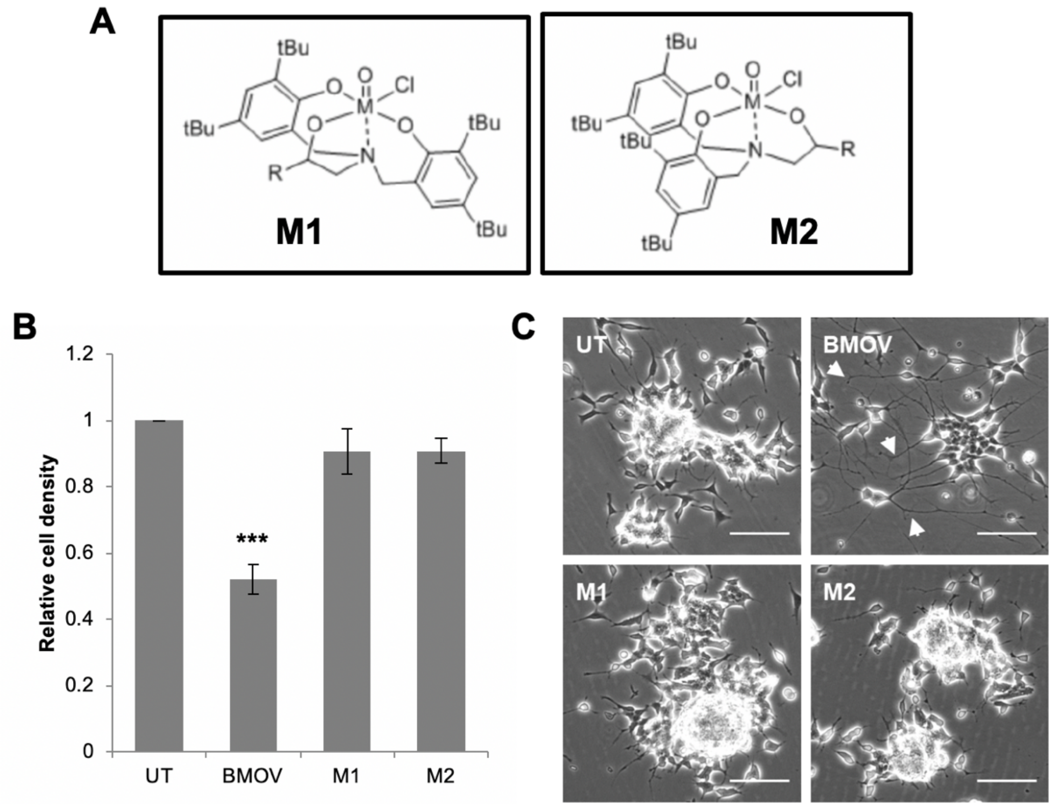

Supplementary Figure S4. Neuroblastoma cells are resistant to molybdenum complexes.

A, M1 and M2 are trans and cis isomer complexes of molybdenum ions centrally co-ordinated with the ligand found in oxidovanadium complex **AL2 (Figure 1)**. **B**, IMR32 cells were treated with 10 µM BMOV, M1 or M2 for 3 days. Cell viability was assessed using crystal violet staining. Reduced cell density was detected after BMOV, but not M1 or M2, treatment. ANOVA with Dunnett post hoc, *** $p<0.001$ (n=3). **C**, SK-N-SH cells were treated with 10 µM BMOV, M1 or M2 for 5 days. Representative images were taken showing that BMOV, but not M1 or M2, induced neurite outgrowth (white arrows). Scale bar = 100 µm.

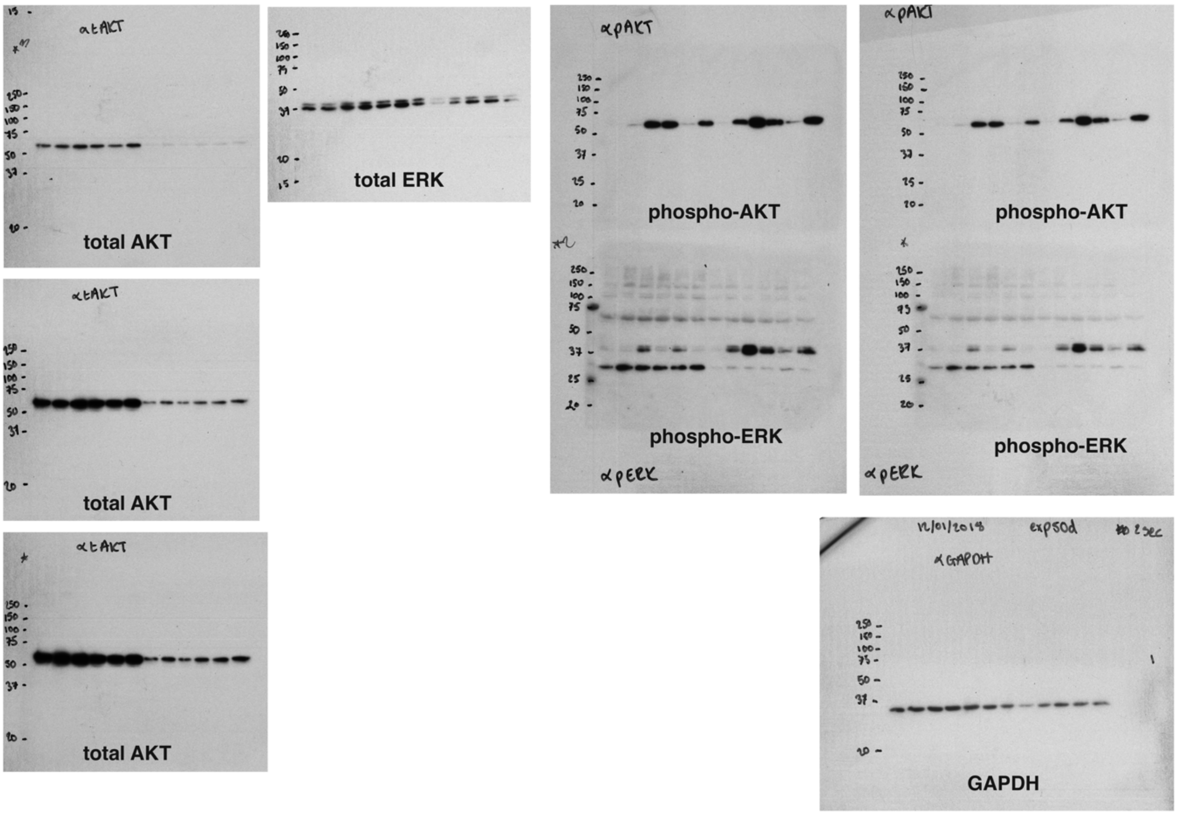

Supplementary Figure S5. Original ECL exposures for panels in Figure 4A

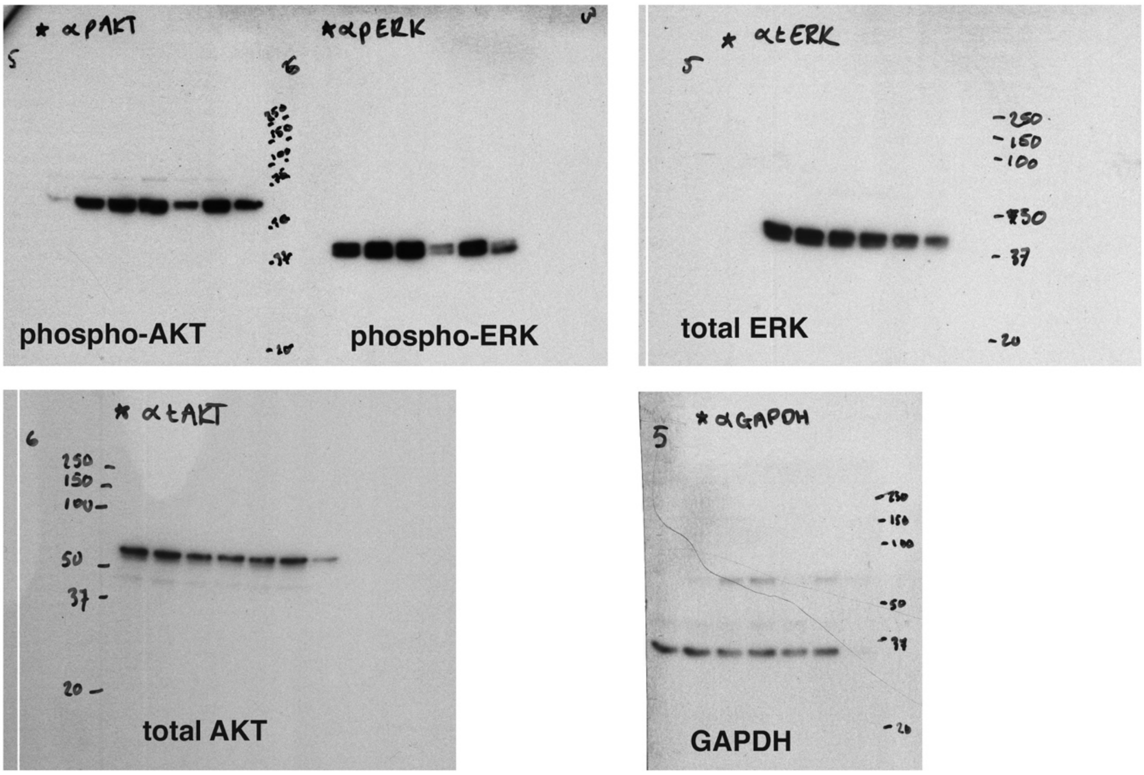

Supplementary Figure S6. Original ECL exposures for panels in Figure 4D

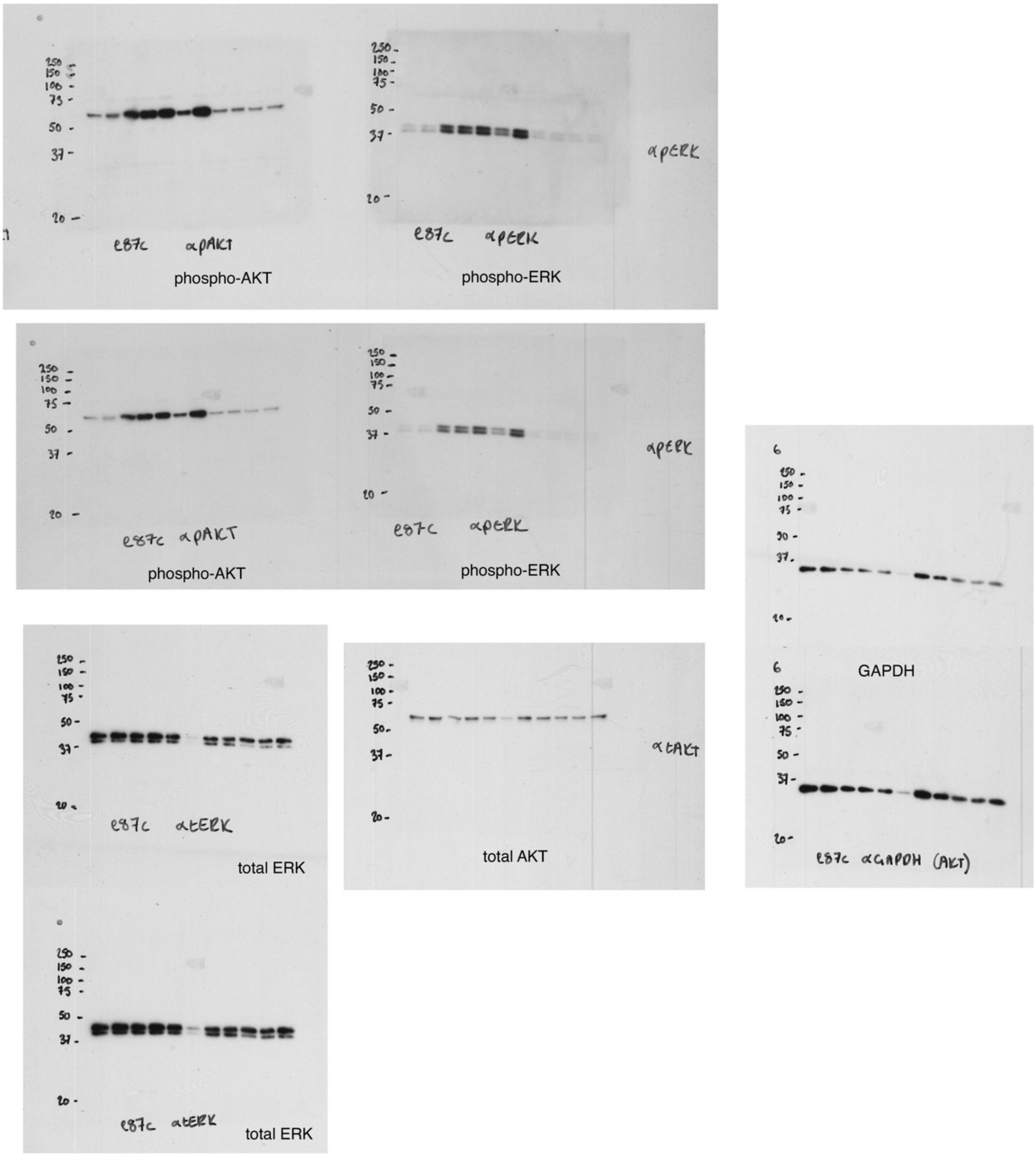

Supplementary Figure S7. Original ECL exposures for panels in Figure 5D

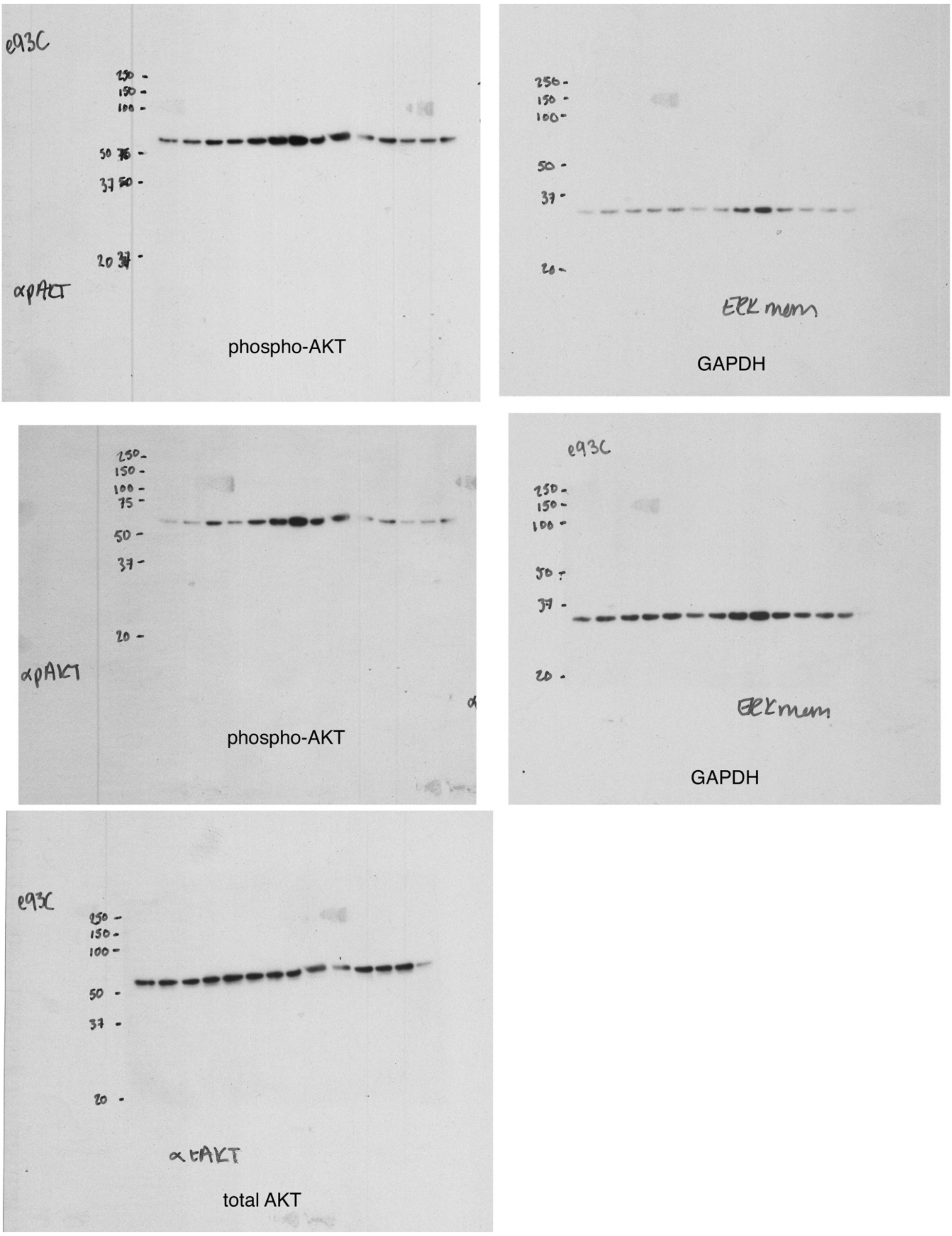

Supplementary Figure S8. Original ECL exposures for panels in Figure 8A

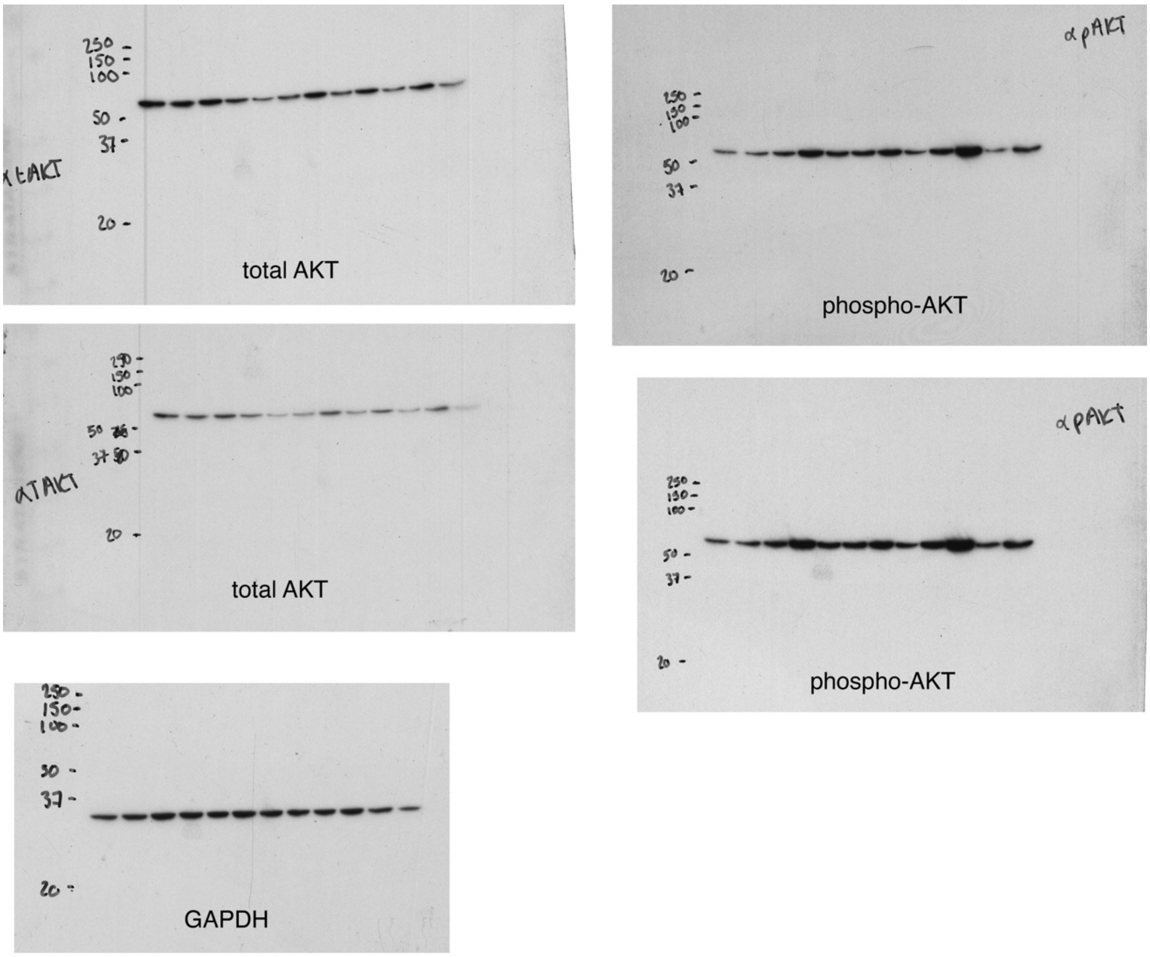

Supplementary Figure S9. Original ECL exposures for panels in Figure 8C
